# Effects of kidney function, serum albumin and hemoglobin on dementia severity in the oldest old people with newly diagnosed Alzheimer’s disease in a residential aged care facility: a cross-sectional study

**DOI:** 10.1186/s12877-020-01789-0

**Published:** 2020-10-07

**Authors:** Jia-Jyun Wu, Shuo-Chun Weng, Chih-Kuang Liang, Chu-Sheng Lin, Tsuo-Hung Lan, Shih-Yi Lin, Yu-Te Lin

**Affiliations:** 1 Center for Geriatrics and Gerontology, Taichung Veterans General Hospital, Taichung, Taiwan; 2 Department of Family Medicine, Taichung Veterans General Hospital, Taichung, Taiwan; 3 Institute of Clinical Medicine, National Yang-Ming University, Taipei, Taiwan; 4 Division of Nephrology, Department of Internal Medicine, Taichung Veterans General Hospital, Taichung, Taiwan; 5 Center for Geriatrics and Gerontology, Division of Neurology, Department of Medicine, Kaohsiung Veterans General Hospital, No.386, Dazhong 1st Rd., Zuoying Dist, Kaohsiung, Taiwan; 6 Tsaotun Psychiatric Center, Ministry of Health and Welfare, Nantou, Taiwan; 7 Department of Psychiatry, School of Medicine, National Yang-Ming University, Taipei, Taiwan; 8 Center for Neuropsychiatric Research, National Health Research Institutes, Miaoli, Taiwan

**Keywords:** Alzheimer’s disease, Clinical dementia rating, Glomerular filtration rate, Mini-mental state examination, Oldest-old

## Abstract

**Background:**

Chronic kidney disease (CKD), low serum albumin, and anemia are known risk factors for cognitive decline in older people. We investigated the association between kidney function and cognitive impairment severity in oldest-old people with a diagnosis of Alzheimer’s disease (AD).

**Methods:**

A cross-sectional study of patients aged 80 years and older was conducted at a veterans’ home in Taiwan between 2012 and 2016. Their estimated glomerular filtration rate (eGFR) was determined using the Modification of Diet in Renal Diseases (MDRD) equation. Cognitive function was evaluated with the Mini-Mental State Examination (MMSE) and Clinical Dementia Rating (CDR).

**Results:**

A total of 84 patients (age mean ± SD, 86.6 ± 3.9 years) had MMSE scores of 10.1 ± 6.7, and CDR scores of 1.6 ± 0.7. The average eGFR was 61.7 ± 21.5 mL/min/1.73m^2^. The mean hemoglobin concentration was 12.7 ± 1.7 g/dl, and the mean albumin concentration was 4.5 ± 4.8 g/dl. Multivariate regression analyses showed that scores of CDR were significantly correlated with eGFR after adjustment for potential confounders. The scores of MMSE were significantly correlated with serum albumin and hemoglobin after adjustment for potential confounders.

**Conclusions:**

We found dementia severity was significantly associated with kidney function, serum albumin, and hemoglobin in the oldest-old with AD. We recommend that oldest-old people with a diagnosis of AD be evaluated to determine kidney function, as well as nutritional and hematological status. Further study is needed to establish whether prevention of CKD deterioration, and correction of malnutrition and anemia may help to slow cognitive decline in oldest-old people with dementia.

## Background

Many epidemiologic studies have found that patients with chronic kidney disease (CKD) are at risk of subsequent cognitive function impairment [1, 2], particularly among the elderly [3]. Furthermore, even in patients with established dementia, it has been shown that renal dysfunction is associated with episodic memory deficits, medial temporal lobe atrophy, and cortical thickness [4]. Several potential mechanisms have been proposed to explain the linkage between CKD and cognitive function decline, including cerebrovascular pathologies, inflammation, alterations in amyloid homeostasis, and metabolic dysregulation [2, 5].

In addition to kidney function, there has been considerable interest in elucidating the role of nutritional factors in impaired cognition and dementia risk among older people. Several studies have reported that low serum level of albumin is independently associated with poor cognitive performance in the elderly [6, 7]. Furthermore, anemia or abnormal hemoglobin concentrations have been reported to be associated with an increased risk for dementia and rapid cognitive decline among the elderly [7, 8].

Individuals aged 80 years and older, who are often termed the “oldest-old” in the literature, are the fastest growing age group in many parts of the world. The prevalence of dementia among the oldest-old population ranges from 18 to 38% [9], and several factors have been proposed to be involved in the development of dementia, including aging, gender, race, education, genotype, diabetes, dyslipidemia, hypertension, and vascular disease [10, 11]. It should be noted that these factors may have slightly different effects on risk of dementia in the very old compared with younger-old subjects. However, the relationship between CKD, nutritional status (biochemical and hematological parameters), and development and/or progression of dementia in the oldest-old is unclear.

In Taiwan, the prevalence of dementia is around 1.7 to 4.3% with the most common type being Alzheimer’s disease (AD) [12]. In a nationwide survey, it was shown that the age-adjusted prevalence of dementia in the following age groups was 3.40% for 65–69 years, 3.46% for 70–74 years, 7.19% for 75–79 years, 13.03% for 80–84 years, 21.92% for 85–89 years, and 36.88% for ≥90 years [12]. Because the oldest-old subpopulation has the highest rate of dementia, which involves a variety of social and financial burdens, provision of satisfactory care for this age group presents clinicians and public health policymakers with a considerable challenge [12, 13]. At present, nearly 70,000 old people (mean age 84.7 years) are living in veterans’ residential communities in Taiwan and are provided with relatively poor psycho-socio-economic resources [14]. Our previous prospective cohort study of the oldest-old with newly diagnosed AD living in a veterans’ home showed that comorbidity burden, as well as nutritional and physical functional status were important predictors of survival [15]. To date, few studies on the factors related to dementia severity, particularly in the oldest-old population, such as kidney function and nutrition, have been conducted in Taiwan. To address this issue, we conducted a cross-sectional study to determine the effect of kidney function, serum albumin, and hemoglobin on dementia severity at the time of AD diagnosis among old people aged ≥80 years.

## Methods

### Study design and population

In Taiwan, older veterans were soldiers that served in World War II and the Chinese Civil War at the end of the 1940s. These older veterans’ gender is often toward male and can choose to live in government-funded assisted living facilities, termed veterans’ homes [16]. At the Jiali Veterans Home in southern Taiwan, the residents with dementia were provided with comprehensive non-pharmacological health services, such as physical exercise, reminiscence activities, spiritual healing, and horticultural therapy to prevent or slow cognitive deterioration. The study consecutively enrolled elderly adults aged at least 80 years old upon admission to the Jiali Veterans’ Home with a probable diagnosis of AD from August 2012 to May 2016. Because all data were based on patients registered in a health system’s dementia database of the Kaohsiung Veterans General Hospital, and analyzed anonymously in a retrospective manner, a verbal or written consent was not required from the enrolled subjects according to the regulations from the ethics committee of the hospital. The study was approved by the ethical review committee conducted by the Institutional Review Boardof Kaohsiung Veterans General Hospital (VGHKS16-CT12–06) and Taichung Veterans General Hospital (CE162332B).

### Diagnosis of dementia

The criteria of the Diagnostic and Statistical Manual IV Edition (DSM-IV) was utilized to establish the diagnosis of dementia. The criteria include the presence of memory impairment, in conjunction with at least one other cognitive deficit (e.g., dysphasia, apraxia, agnosia, or impairments in judgement or abstract thinking, personality changes or constructional difficulties). These features represented declines from a formerly better functionality to sufficient magnitudes of impairment, which impacted the social and occupational realms of the patients. It took a comprehensive history review, neurological examination, laboratory survey, and neuroimaging study to establish a diagnosis of probable AD clinically. All patients met the diagnostic criteria for probable AD, as proposed in 1984 by the workgroup of the National Institute of Neurological and Communicative Disorders and Stroke and the AD and Related Disorders Association (NINCDS-ADRDA) [17].

### Measurement of kidney function

Blood samples for measuring serum creatinine levels were obtained during the patient’s first visit. The estimated Glomerular Filtration Rate (eGFR) was ascertained exactly using the formula of the Modification of Diet in Renal Disease (MDRD) equation for Taiwanese adults [18]. We chose the MDRD equation for the present study because a previous study of an oldest-old population found that this equation best-predicted mortality when eGFR was between 45 and 59 mL/min/1.73m^2^ [19]. Although it remains controversial as to which eGFR threshold should be referred to determine CKD in geriatrics, CKD has frequently been specified as an eGFR< 60 ml/min/1.73m^2^ for at least 3 months, in accordance with the Kidney Disease Improving Global Outcome (KDIGO) Clinical Practice Guideline, as self-reported by the patients or retrieved from electronic records.

### Measurement of cognitive function

The Mini-Mental State Examination (MMSE) provides adequate assessment of the severity of cognitive dysfunction, which varies within the population by age and education [20]. The MMSE is a 30-point questionnaire that includes tests of orientation, attention, memory, and language, and higher values indicate a superior level of functioning.

The Clinical Dementia Rating scale (CDR) ranges from 0 to 3 points, and is used to identify cognitive domains and functional performance for AD and related dementias. The cognitive performance is rated in testing 6 cognitive and behavioral domains consisting of memory, orientation, judgment and problem solving, community affairs, home and hobbies, and personal care. It is completed by interviewing with the patient and a reliable main caregiver. The global CDR score is a weighted average of the category ratings, where a value of 0 represents no dementia, a value of 0.5 appears for questionable dementia (with a cognitive decline but not satisfying the criteria for dementia), and scale 1, 2, and 3 stands for mild, moderate, and severe dementia, respectively [21].

### Determination of potential risk factors

Potential risk factors that were examined included demographics, lifestyle, and comorbid illness. Demographic variables were age, gender, and education. Lifestyle variables were tobacco smoking, and alcohol drinking. Similarly, we also performed blood biochemistry analyses to determine glucose, hemoglobin, albumin, liver function, triglycerides, and total cholesterol. In Taiwan, the National Health Insurance program, which was established in 1995, provides comprehensive health insurance coverage to over more than 99% of the island’s 23 million people [22]. Accordingly, comorbidities such as diabetes, hypertension, cardiovascular disease, etc., were reviewed by codes of the international classification of diseases or medications treated for the diseases in the medical record. The total number of medications used was also recorded. The activities of daily living scale (ADL) were determined by the Barthel index (a scale between 0 and 100) to assess the patients’ functional status. All of the variables listed were potential confounders and thus warranted inclusion in the statistical models examining renal function and cognitive performance.

### Statistical methods

Quantitative data were expressed by mean values ± standard deviations (SD). Categorical data were expressed as numbers with percentages. The quantitative comparison across groups was checked by the Mann-Whitney U test, while the chi-square test/Fisher’s exact test was used for categorical ones. Before correlation analyses, continuous variables (MMSE and CDR) were log-transformed from skewed to normal distributions. The correlation between CDR, MMSE scores, and various parameters was calculated using the Spearman’s rank correlation test. Finally, simple and multiple linear analyses were used to assess the relationship between the cognitive assessment and various clinical data. A two-tailed *p*-value was considered to be statistically significant if it was less than 0.05. All analyses were performed using SPSS for Windows version 16.0 (SPSS Institute Inc., Chicago, IL, USA).

## Results

### Baseline characteristics

In total, 84 patients diagnosed with AD were enrolled in the study, of whom 78 were male (92.9%), with a mean age of 86.6 ± 3.9 years. Over half (56.0%) had no formal education or had a total education period of less than 6 years. The mean number of comorbidities was 2.3 ± 1.4, and the mean number of drugs taken was 4.7 ± 3.0. The overall MMSE scores were 10.1 ± 6.7 and CDR scores were 1.6 ± 0.7 (Table 1). Forty-four patients (52.4%) had a CDR score equal to or higher than 2, indicating a stage of moderate to severe dementia. The average eGFR was 61.7 ± 21.5 mL/min/1.73m^2^ with 50.0% eGFR levels ≥60 mL/min/1.73m^2^, and 25.0% eGFR levels between 45 and 60 mL/min/1.73m^2^, as well as 25.0% eGFR levels < 45 mL/min/1.73m^2^. CKD was found in 50.0% of the patients (Table 1). The mean hemoglobin concentration was 12.7 ± 1.7 g/dl, and mean serum albumin concentration was 4.5 ± 4.8 g/dl.

**Table 1:** Baseline demographic and clinical characteristics of patients

Total number of patients	*n* = 84
Age (years)	86.6 ± 3.9
Men, *n* (%)	78 (92.9)
Education (> 6 years),*n* (%)	37 (44.0)
Number of medications, mean (SD)	4.7 ± 3.0
Lifestyle behaviors, *n* (%)	
Tobacco smoking habit	30 (35.7)
Alcohol drinking habit	19 (22.6)
Comorbidities, *n* (%)	
Hypertension	51 (60.7)
Cardiovascular disease	18 (21.4)
Diabetes mellitus	15 (17.9)
Cerebral vascular disease	13 (15.5)
Number of comorbidities, mean (SD)	2.3 ± 1.4
Geriatric assessment, mean (SD)	
MMSE	10.1 ± 6.7
CDR	1.6 ± 0.7
ADL	68.8 ± 28.5
GDS	3.2 ± 3.8
Laboratory data, mean (SD)	
Creatinine (mg/dL)	1.2 ± 0.4
eGFR (mL/min/1.73m^2^)	61.7 ± 21.5
Fasting glucose (mg/dL)	95.2 ± 28.0
Triglyceride (mg/dL)	142.1 ± 62.4
Cholesterol (mg/dL)	146.3 ± 50.5
Albumin (g/dL)	4.5 ± 4.8
GPT (U/L)	14.9 ± 7.3
White blood cell (/mm^3^)	6136.6 ± 2070.4
Hemoglobin (g/dL)	12.7 ± 1.7
Vitamin B12 (pg/mL)	409.9 ± 223.3
TSH (μIU/mL)	1.6 ± 1.3
MDRD eGFR (mL/min/1.73m^2^),*n* (%)	
eGFR≥60	42 (50.0)
eGFR45–59.99	21 (25.0)
eGFR< 45	21 (25.0)

*eGFR* Estimated glomerular filtration rate, calculated by MDRD; *MMSE* Mini-mental state examination; *CDR* Clinical Dementia Rating Scale; *ADL* Activities of daily living; *GDS* Geriatric depression scale; *GPT* Glutamic pyruvic transaminase; *TSH* Thyroid-stimulating hormone; number of comorbidities (comorbidities included depression, hypertension, cardiovascular disease, diabetes mellitus, cerebral vascular disease, lung diseases, kidney diseases, liver diseases, gastrointestinal diseases, and genitourinary diseases)

### Comparison of eGFR and cognitive function

Compared to those without CKD, participants with CKD had lower MMSE scores and higher CDR scores, although the differences in CDR were not statistically significant (Table 2). Results of simple linear analyses by Spearman’s correlation showed a significant relationship between eGFR, MMSE, and CDR scores (Table 3). Prior to regression analyses, continuous variables (MMSE and CDR) were also log-transformed from skewed to normal distributions. The relationship between eGFR levels and scores of CDR (but not MMSE) was still statistically significant after adjustment for potential confounding variables (Tables 4 and 5). In addition, there was a significant association between ADL scale and CDR scores (Table 5).

**Table 2:** MMSE and CDR scores between various categories

	MMSE		*P*-value	CDR		*P*-value
	Mean	±SD		Mean	±SD	
Gender			0.763			0.763
Men	10.1	±6.6		1.6	±0.8	
Women	9.8	±8.7		1.7	±0.5	
Education (yrs)			0.305			0.385
0–6 years	10.5	±6.8		1.6	±0.7	
> 6 years	9.6	±6.7		1.7	±0.7	
Number of medications			0.458			0.719
0–4	9.22	±5.89		1.59	±0.78	
≥ 5	10.90	±7.67		1.63	±0.70	
Number of comorbidities			0.359			0.551
0–1	8.62	±5.37		1.70	±0.75	
≥ 2	10.51	±7.11		1.58	±0.73	
MDRD eGFR^a^			0.012			0.112
eGFR≥60	12.6	±7.3		1.5	±0.8	
eGFR45–59.99	9.1	±6.6		1.6	±0.8	
eGFR< 45	6.7	±5.1		1.8	±0.4	
Tobacco smoking habit			0.364			0.512
No	9.4	±6.3		1.7	±0.8	
Yes	11.1	±7.4		1.5	±0.6	
Alcohol drinking habit			0.290			0.817
No	9.6	±6.8		1.6	±0.8	
Yes	11.6	±6.4		1.6	±0.6	
Hypertension			0.001			0.216
No	6.4	±5.2		1.8	±0.7	
Yes	12.0	±6.7		1.6	±0.7	
Cardiovascular disease			0.972			0.786
No	10.1	±7.0		1.6	±0.7	
Yes	10.0	±5.9		1.6	±0.8	
Diabetes mellitus			0.322			0.932
No	10.5	±6.8		1.6	±0.8	
Yes	8.3	±6.5		1.6	±0.6	
Cerebral vascular disease			0.011			0.079
No	9.2	±6.3		1.7	±0.7	
Yes	15.9	±6.7		1.3	±0.9	

Mann-Whitney U test. ^a^Kruskal Wallis test

**Table 3:** Correlation of MMSE and CDR with other measured variables

	MMSE (log)		CDR (log)	
	r	*P-*value	r	*P-*value
Age	−0.09	0.465	−0.06	0.632
Number of comorbidities	0.17	0.169	−0.17	0.131
Number of medications	−0.07	0.593	0.04	0.744
ADL	0.12	0.327	−0.34	0.002**
Laboratory data				
Creatinine (mg/dL)	−0.22	0.078	0.25	0.041*
eGFR (mL/min/1.73m^2^)	0.32	0.009**	−0.30	0.013*
Fasting glucose (mg/dL)	−0.09	0.468	0.08	0.501
Triglyceride (mg/dL)	0.12	0.343	−0.19	0.115
Cholesterol (mg/dL)	−0.25	0.044*	0.35	0.003**
Albumin (g/dL)	−0.30	0.028*	−0.15	0.246
GPT (U/L)	0.20	0.181	−0.12	0.398
White blood cell (/mm3)	−0.06	0.635	0.17	0.134
Hemoglobin (g/dL)	0.33	0.006**	−0.17	0.132
Vitamin B12 (pg/mL)	−0.10	0.396	−0.01	0.952
TSH (μIU/mL)	−0.04	0.743	−0.05	0.662

*P-value < 0.05, **P-value < 0.01

**Table 4:** Univariate and multivariate analysis of MMSE (log) predictors

	Simple linear regression			Multiple linear regression		
	B	Beta(β)	*P-*value	B	Beta(β)	*P-*value
Age	−0.01	−0.09	0.465			
Gender (F vs M)	−0.0004	0.00	0.998			
Education (> 6 vs ≦6 years)	−0.05	− 0.06	0.637			
Number of medications	−0.01	−0.07	0.593			
Number of comorbidities	0.05	0.17	0.159			
Hypertension	0.35	0.39	0.001**	0.26	0.30	0.017*
Cardiovascular disease	0.03	0.03	0.801			
Diabetes mellitus	−0.15	−0.14	0.255			
Cerebral vascular disease	0.34	0.27	0.022*	0.15	0.12	0.350
ADL	0.00	0.12	0.327			
Creatinine (mg/dL)	−0.21	−0.22	0.078			
eGFR (mL/min/1.73m^2^)	0.01	0.32	0.009**	0.003	0.14	0.285
Cholesterol (mg/dL)	−0.002	−0.25	0.044*			
Albumin (g/dL)	−0.02	− 0.30	0.028*	− 0.03	−0.34	0.008**
Hemoglobin (g/dL)	0.08	0.33	0.006**	0.07	0.29	0.019*

Multiple linear regression (Stepwise). *P-value < 0.05, **P-value < 0.01

**Table 5:** Univariate and multivariate analysis of CDR (log) predictors

	Simple linear regression			Multiple linear regression		
	B	Beta(β)	*P-*value	B	Beta(β)	*P-*value
Age	−0.003	−0.06	0.632			
Gender (F vs M)	0.05	0.08	0.505			
Education (> 6 vs ≦6 years)	0.01	0.03	0.820			
Number of medications	0.00	0.04	0.744			
Number of comorbidities	−0.03	−0.19	0.107			
Hypertension	−0.05	− 0.14	0.212			
Cardiovascular disease	−0.01	−0.03	0.785			
Diabetes mellitus	0.004	0.01	0.940			
Cerebral vascular disease	−0.09	−0.18	0.117			
ADL	−0.002	−0.34	0.002**	−0.001	− 0.24	0.026*
Creatinine (mg/dL)	0.09	0.25	0.041*			
eGFR (mL/min/1.73m^2^)	−0.002	−0.30	0.013*	−0.002	− 0.30	0.014*
Cholesterol (mg/dL)	0.001	0.35	0.003**			
Albumin (g/dL)	−0.01	−0.15	0.246			
Hemoglobin (g/dL)	−0.02	− 0.17	0.132			

Multiple linear regression (Stepwise). *P-value < 0.05, **P-value < 0.01

### Associations of serum albumin and hemoglobin with cognitive function

The Spearman’s rank correlation test showed a statistically significant positive correlation between MMSE scores and hemoglobin and albumin levels (Table 3). The relationships of albumin and hemoglobin values with scores of MMSE (but not CDR) were still statistically significant after adjustment for potential confounding variables (Tables 4). Besides, a history of hypertension was also shown to be independently associated with MMSE scores.

### Associations of numbers of comorbidities and medications used with cognitive function

It was shown that there was no relationship between number of comorbidities, medications and MMSE, and CDR scores, respectively, by Spearman’s correlation (Table 3), and univariate regression analysis (Tables 4 and 5).

## Discussion

In this cross-sectional study, we found that in the oldest old patients with AD, eGFR was significantly associated with dementia severity scores by CDR. In addition, serum albumin and hemoglobin were significantly associated with MMSE scores. These results suggest that renal, nutritional, and hematological status may play a role in disease progression in the oldest old patients with dementia.

The prevalence of CKD increases markedly with age. An analysis of the 30,528 participants in the NHANES 1988–1994 and 1999–2006 pooled cohort revealed that the highest prevalence of eGFR < 60 mL/min/1.73m^2^ was among those ≥80 years of age, with a rate of nearly 51% [23]. In our study, the percentage of oldest old with CKD was 50%, which was compatible with the aforementioned report. Several studies have shown that risk of cognitive decline or dementia is increased in older adults with reduced kidney function [3]. However, only a few studies have investigated the association between the decrease in renal function and cognitive performance in the oldest old people. Three previous studies reported that eGFR was associated with cognitive impairment in individuals aged 80 years or older [24–26]. Our results were in line with the aforementioned studies, and also found a significant association between impaired kidney function and cognitive decline in the oldest old people, although some other studies showed conflicting results [27, 28]. The reason that eGFR was associated with CDR but not MMSE scores after adjustment for potential confounding variables is not clear. It has been reported that most domains of cognitive function are affected by impaired kidney function across all stages of CKD with the executive function being affected earlier than episodic memory and global ability, which may be assessed by CDR [3]. In contrast, MMSE is limited in the assessment of all cognitive domains, with the notable absence of executive function and psychomotor speed [29].

It was proposed that CKD in old people may often coexist with risk factors for cognitive impairment, such as diabetes, hyperlipidemia, and cardiovascular disease, and these in turn can contribute to the development of dementia [27]. In addition, neuroimaging studies showed that kidney function is associated with hippocampal volume, white matter hyperintensity, and cognitive decline in oldest old patients with mild cognitive impairment and AD [10, 30, 31]. Several pathological changes, such as hippocampal sclerosis, amyloid angiopathy, and microvascular injury in particular, have been found in the oldest old dementia patients, including Alzheimer’s disease [32]. Because CKD is often associated with systemic endothelial injury, it may contribute to the development of dementia by interacting with neuronal pathologies [33]. Finally, in renal insufficiency, amyloid homeostasis may be altered, which thus exacerbates neuronal damage [34]. Overall, previous studies in the literature as well as the investigation presented herein indicate a relationship between kidney function and cognition decline in the oldest old individuals with dementia.

CKD in the older population has been associated with several CKD-related metabolic complications, such as anemia and hypoalbuminemia [23]. In our study, after adjustment for kidney function, serum albumin and hemoglobin were both still associated with MMSE scores, suggesting their potential roles in the development of AD in the oldest old. This association of cognitive impairment with serum albumin and hemoglobin levels may be explained by certain pathophysiological mechanisms. Albumin is an antioxidant which may help prevent excessive oxidant stress induced by inflammation in the aging neuronal cell [35]. As inflammatory mechanisms are involved in the pathogenesis of dementia, including Alzheimer’s disease [36], low serum albumin levels may become a risk factor for cognitive decline in AD. Anemia can cause tissue oxygenation, and consequently reduce the reserve response of the brain to external insults, and promote neuronal degeneration [37]. Thus, low hemoglobin levels may potentially predispose to dementia and poor cognitive performance. Also, both hypoalbuminemia and anemia may be a consequence of underlying comorbidities, and nutrient deficiency (e.g., folate and vitamin B12), which have been shown to have deleterious effects on cognitive functioning [35, 38]. However, it should be noted that MMSE scores are influenced by several factors such as age, educational level, and premorbid intelligence of the patients [20, 21]. Further longer studies are required to clarify the relationship between serum albumin, hemoglobin, and disease severity in the oldest old people with dementia. A variety of risk factors for cardiovascular disease are reported to have less obvious effects on cognition in the oldest old when compared with younger individuals [39, 40]. In individuals over 75 years of age, it was reported that high systolic blood pressure (≥ 160 mmHg) was not associated with a greater risk of dementia [41], and the risk of dementia even decreased with an increasing blood pressure level in subjects aged 85 years or older [42]. In our study, we found that in the oldest old, those with a history of dementia and hypertension tended to exhibit less decline in cognition. This finding supports previous reports, although the optimal blood pressure with respect to dementia risk in this subpopulation requires further research. In this study, we also found that ADL scale was significantly associated with dementia severity scores by CDR. This finding was compatible with several previous reports that cognitive decline affects the performance of activities of daily living in patients with dementia [43]. Executive dysfunction is a common manifestation of AD in all stages, and it has been shown in several studies that there is a link between executive dysfunction and impaired performance on ADL scales [44]. The effect of cognitive functions on changes of daily functions is important because it will tell clinicians and families to provide appropriate interventions to improve or maintain daily performances for preparation of sufficient supporting resources in the care of older patients with dementia.

In a previous study in Taiwan, it was demonstrated that the risk of dementia increased with the number of medications used, possibly due to their adverse effects on the central nervous system [45]. Besides, multiple comorbidities also exhibited a strong influence on dementia. Although, in our study, the numbers of comorbidities and medications were not related to dementia severity at the time of AD diagnosis, careful management of comorbidities and medications is necessary to prevent dementia. On the other hand, people with dementia often present with concomitant chronic medical conditions, that may worsen clinical course (i.e., by accelerating cognitive and functional decline) and complicate their pharmacological management for dementia [46]. Thus, after the diagnosis of dementia, a comprehensive evaluation of associated diseases and all pharmacological treatments was required [46].

A few demographic and social factors have been identified as risk factors for developing dementia in the oldest old, including the pre-dementia status of instrumental activities during daily living, mental stimulation, and levels of leisure activities [47]. In our study, it was shown that moderate to severe dementia by CDR was present in 51.8% of the subjects who were newly admitted to the dementia care institute. Two previous reports have shown similar severity rates in residents at nursing homes [48, 49], although some have reported less severe rates [50, 51]. We speculate that our oldest old patients’ symptoms of dementia might not have been recognized early because their disability may have been attributed to comorbid diseases or other physiological complexities, rather than to dementia. In our patients, the majority (56.0%) had less than 6 years of education, and there was no significant association between education level and dementia severity by CRD or MMSE scoring at the diagnosis of AD. It has been reported that education is only a relevant variable for understanding cognitive performance in older age, as it is related to the rate of decline in aging [52]. Moreover, some studies have found education to be a risk factor for vascular dementia, rather than Alzheimer’s disease [53].

Our findings may have some clinical implications. A recent study reported that among frail elderly individuals’ severity of renal dysfunction was independently correlated with cognitive impairment [26]. It was suggested that a combination of cognitive and renal function decline may more exacerbate the vulnerability of older persons, and resulted in adverse health-related outcomes. Altogether, our study findings as well as the previous report implicate future research focusing efforts on identifying renal impairment early in patients with dementia to prevent frailty progression and preserve the quality of life. Besides, some experimental and clinical studies have shown that through inhibiting the renin-angiotensin system in patients with dementia, not only is the progression of renal disease slowed down, but dementia incident risks can also be reduced [54, 55]. Moreover, restoration of malnutrition and anemia has also been reported to improve cognition in older patients with dementia [56, 57]. In caring for elderly patients with dementia, clinicians should not only recognize the severity of cognitive dysfunction, but should also examine the nutrition and hemoglobin before proposing treatment strategies to slow progression of cognition impairment.

There were several limitations in this study. First, it employed a cross-sectional design, and it lacked a control group. Thus, a causal relationship could not be established. Second, the study population was small, and more than half of the enrolled subjects were at a moderate or severe stage of cognition impairment. Based on the findings presented herein, it is not possible to establish whether the associations between renal and cognitive function would be found among oldest old patients in the early stage of their disease, and thus further investigation is required. Third, about 93% of the study subjects were male. Thus, the result may be hard to be representative of both genders. Because many studies have reported that women have a higher incidence rate of dementia than men especially at their oldest-old ages [9]. Further research in the oldest-old female patients with dementia is necessary to establish a more definite conclusion. Fourth, using CKD-MDRD eGFR to represent renal function in oldest old individuals may not be accurate as no consensus has been reached regarding the best renal assessment method for this age subpopulation [58]. Lastly, other potential risk factors for oldest old dementia, such as low level of physical activity, depression, delirium, inflammatory markers, genotyping, and drugs for AD treatment were not examined in the current study [9–11]. Further larger and longitudinal analyses are required to determine whether decreased renal function, nutritional, and hematological status predict cognitive decline in the oldest old with dementia.

## Conclusions

We demonstrated that severity of cognitive decline was correlated with eGFR, serum albumin, and hemoglobin in the oldest old patients diagnosed with probable AD. Therefore, in addition to evaluating traditional risk factors known to be associated with cognition function (e.g., cardiovascular risk factors), we recommend that renal function, as well as biochemical and hematological status should also be assessed. Whether the prevention of kidney function deterioration, and restoration of albumin and hemoglobin may help to slow or halt cognitive deterioration in oldest old patients with dementia requires further study.

## Data Availability

The datasets used and analyzed during the current study are available from the corresponding author on reasonable request.

## Abbreviations

CKD: Chronic kidney disease
AD: Alzheimer’s disease
eGFR: Estimated glomerular filtration rate
MDRD: Modification of diet in renal diseases
MMSE: Mini-mental state examination
CDR: Clinical dementia rating
DSM-IV: Diagnostic and statistical manual IV edition
NINCDS-ADRDA: National institute of neurological and communicative disorders and stroke and the AD and related disorders association
KDIGO: Kidney disease improving global outcome
ADL: Activities of daily living
SD: Standard deviation
